# Development of an integrative coding framework for evaluating context within implementation science

**DOI:** 10.1186/s12874-020-01044-5

**Published:** 2020-06-15

**Authors:** L. Rogers, A. De Brún, E. McAuliffe

**Affiliations:** University College Dublin Centre for Interdisciplinary Research, Education and Innovation in Health Systems (UCD IRIS), UCD School of Nursing, Midwifery and Health Systems, Dublin 4, Ireland

**Keywords:** Context, Contextual factors, Healthcare, Implementation science, Evaluation

## Abstract

**Background:**

This research aims to explore an identified gap in implementation science methodology, that is, how to assess context in implementation research. Context is among the strongest influences on implementation success but is a construct that is poorly understood and reported within the literature. Consequently, there is little guidance on how to research context. This study addresses this issue by developing a method to account for the active role of context during implementation research. Through use of a case study, this paper demonstrates the value of using our context coding framework.

**Methods:**

The developed context coding framework was guided by the sub-elements of the Consolidated Framework for Implementation Research (CFIR). Employing a constructivist approach, this framework builds on the CFIR and enables a deeper exploration of context at multiple levels of the health system. The coding framework enables the collation of various data sources such as organisational reports, culture audits, interview, survey, and observational data. It may be continuously updated as new data emerge and can be adapted by researchers as required. A pre-existing rating criterion has been integrated to the context coding framework to highlight the influence and relative strength of each contextual factor prior to and during implementation.

**Results:**

It is anticipated that the context coding framework will facilitate a standardised approach to assessing context. This will provide a deeper understanding of how to account for the influence of context, ultimately providing guidance that should increase the likelihood of implementation success. The coding framework enables implementation progress to be monitored, facilitating the identification of contextual changes and variations across settings at different levels of the healthcare system. It is expected this framework will inform the selection of appropriate implementation strategies and enable the monitoring of such strategies regarding their impact on local context.

**Conclusions:**

This research contributes to the extant literature by advancing methodologies for the consideration and assessment of context in implementation research. This context coding framework may be used in any setting to provide insight into the characteristics of particular contexts throughout implementation processes.

## Background

It is widely acknowledged that the uptake of evidence-based healthcare interventions is challenging [1–4] with it being reported that only 50–60% of care in the past decade has been delivered in line with the best available evidence [5]. It is suggested that this is due to a naïve assumption that the implementation of a new intervention will be self-evident, disseminating automatically through a linear *‘pipeline model’* whereby an intervention moves from the laboratory, into an effectiveness trial before sustained application in routine practice [4, 6, 7]. However, this simplistic model does not account for the complexity associated with health systems. Context has been cited as a key concept impacting the translation of evidence into practice [8–11] and accounting for it is critical in interpreting and generalising findings [12–14]. Yet despite this noted importance, context is often overlooked by researchers within the field of implementation science which has led to an insufficient understanding of this construct.

This poor understanding has been attributed to the variability in how context is defined and assessed across studies [6, 15–20]. However, Rogers et al.’s [20] systematic review confirms that inconsistencies exist in the extant literature when defining context. This review also identifies commonalities across the papers included to develop a broad operational definition for the construct. Using a complexity science lens, this conceptualisation recognises the interconnections of system components, defining context as

“…a multi-dimensional construct encompassing micro, meso and macro level determinants that are pre-existing, dynamic and emergent throughout the implementation process. These factors are inextricably intertwined, incorporating multi-level concepts such as culture, leadership, and the availability of resources”*.*

Buttney [21] suggests that *“we need to take context as that which can be empirically investigated rather than some factor that is a priori assumed to be at work”.* To address this gap, a method to adequately account for context within implementation research is necessary This is echoed by Murdoch [22] and Fernandez et al. [23] who argue that understanding the dynamics of context requires its conceptualisation to be translated into a practical method of assessment. However, Rogers et al.’s [20] systematic review observed considerable heterogeneity in the approaches used to assess context. Over 40 methods were identified within the 64 papers included which led to the recommendation that a standardised approach for assessing context is required integrating a qualitative method informed by a comprehensive framework. This approach is considered the most suitable to ensure an in-depth assessment of context is achieved.

To address this identified gap, a context coding framework has been developed. This paper outlines how the context coding framework was created and highlights the value of employing this approach within implementation science research, using an illustrative case study to demonstrate its application.

## Methods

### Study background

This study is part of a wider body of research that uses a case study design to investigate the active role of context during the implementation of a healthcare initiative. The introduction of a collective leadership intervention was chosen as the implementation case study and is the primary focus of this research. This intervention aims to introduce collective leadership to healthcare teams using a suite of interventions to improve team performance and safety culture [24]. The collective leadership interventions have been piloted over a one-year period with four heterogeneous healthcare teams. The results presented in this paper incorporate data from one of the four participating teams as an illustrative case study to highlight the value of applying the context coding framework during the implementation of a healthcare initiative. Characteristics of the chosen case are summarised in Table 1.

**Table 1:** Description of Case A

Case A	
Hospital classification	Model 3- Hospitals that can provide 24-h acute surgery, acute medicine, and critical care.
Location	Rural
Financial Structure	Statutory hospital
Hospital size	Approximately 200 bed capacity
Team size	*n* = 65
Team Specialty	Surgical

### Measures

As outlined in the introduction, contextual conditions are the pre-existing, dynamic and emergent factors of implementation that encompass multi-level determinants. To gain a greater understanding of implementation, it is essential to monitor these contextual conditions in research as the data generated is context dependent. Literature suggests that theories, models and frameworks provide greater insight into the mechanisms of implementation [15, 25]. The Consolidated Framework for Implementation Research (CFIR) is one of the most widely operationalised frameworks that aids in the characterisation of contextual determinants [23, 26–28]. Given the comprehensive nature of the CFIR and the vast quantity of frameworks available within the field of implementation science [27], CFIR was chosen to inform the development of the context coding framework. By synthesising overlapping constructs, the CFIR provides a repository of implementation related concepts which are organised into five major domains; intervention characteristics, outer setting, inner setting, individual characteristics and the implementation process [29].

**Fig. 1:**
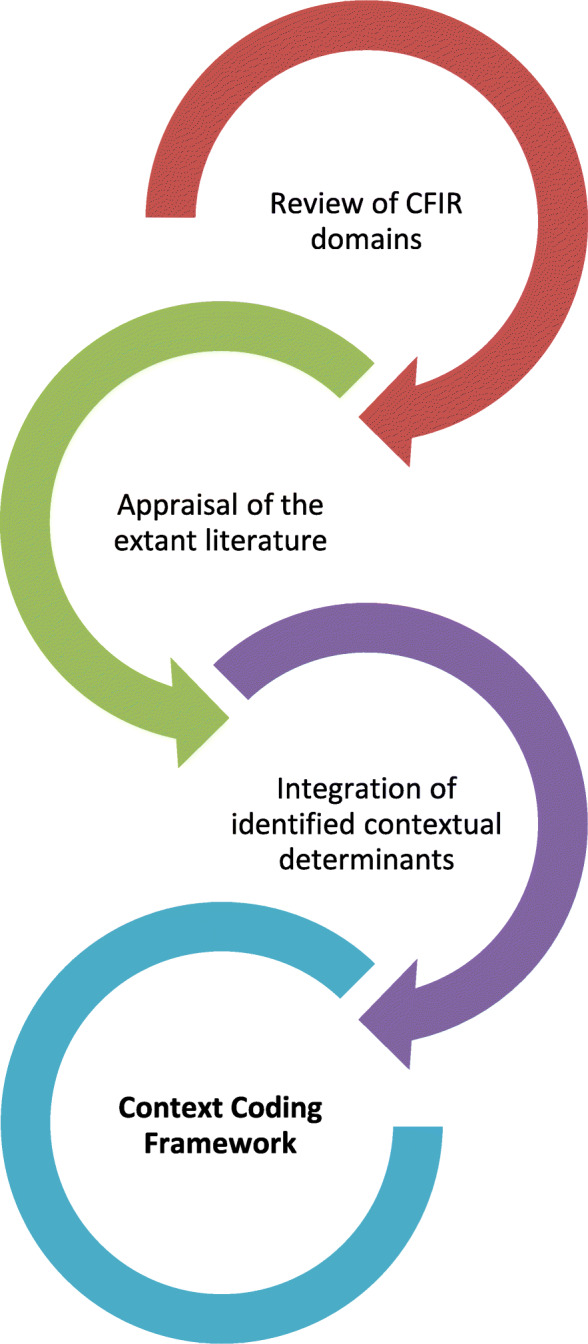
Context coding framework development

Using a constructivist approach, the context coding framework builds on the CFIR to enable a deeper exploration of contextual factors independent of the intervention. By grounding the analysis across system levels, the framework accounts for team-level contextual factors previously overlooked in implementation science [20] and rearranges CFIR determinants to the appropriate system level (individual, team, organisational, and system levels) (Table 2). Figure 1 outlines the process which identified contextual factors relating to these system levels. Firstly, all five CFIR domains were reviewed to determine the applicability of each construct in describing the surrounding context. For example, the relative advantage construct within the *Intervention characteristics* domain can outline improvements in the surrounding context which participants attribute to the intervention. Within the context coding framework this CFIR construct is depicted as the individual-level determinant *individual attitudes* (Table 2)*.* Next, the extant literature was appraised to collate contextual determinants not explicitly referenced by the CFIR [20, 29–36]. Rogers et al.’s [20] recent review provided a diverse range of novel constructs including team-level contextual determinants (Table 2). Finally, the contextual determinants of CFIR were integrated with the findings from the literature to refine the established CFIR constructs. For example, leadership engagement within the *Inner setting* domain was revised to highlight the influence of leadership at multiple system levels; team (local leadership engagement), organisational (organisational leadership engagement), and system (political environment) (Table 2). Subsequently the context coding framework provides a revised collection of contextual features that are pertinent to implementation and maps these determinants to the micro, meso and macro levels of the health system (Table 2).

**Table 2:** Context Coding Framework

System Level	Characteristic	Definition		Example
System-Level Determinants	Social Environment	Cosmopolitanism	How connected the hospital is with external organisations/events and the impact of this network?	View that being affiliated with a hospital group (hospitals in Ireland organised into seven hospital groups) provides more learning opportunities for staff.
		Peer Pressure	Mimetic or competitive pressure to implement an intervention.	A team member asks researchers if other teams have “embedded it better” and how this was achieved.
	Political Environment	External Incentives and Influence	External incentives to spread the uptake of interventions (national policy, guidelines, collaborations), external influence regarding decision making (e.g. external change agents).	Perceived threat following a proposed systems change leaving staff anxious about future prospects.
	Economic Environment	External economic factors within the wider health system which may influence the capacity and resources available to the setting.		Disparity in funding. Hospitals, comparable in size and characteristics acknowledged as receiving greater resources due to previously publicised incidents.
Organisational-Level Determinants	Structural characteristics	Hospital Classification		Participants confirm an increased demand on the hospital, with the number of patients on trolleys exceeding the norm.
		Hospital size		
		Hospital workload		
	Networks and communications	The quality of communication within the organisation and relationships amongst its members.		National survey data highlight strong relationships among staff, however, relationships between management and frontline appear taut.
	Culture	The norms, values and assumptions of the organisation, the degree of autonomy given to staff and their perceptions of change.		Hospital documents suggest a culture characterised by openness, trust and inclusion.
	Compatibility	Is there a tangible fit between the values and norms of the organisation to the intervention?		The collective leadership intervention appears to align with the open culture outlined in hospital reports.
	Organisational support	Is organisational support evident? Are rewards offered by the organisation for engagement with the intervention?		Food provided by the organisation at each session and is suggested to enhance staff attendance.
	Organisational climate	Staff perceptions of and emotional responses to the characteristics of their organisation including attitudes towards learning.		One team member discusses the importance of valuing staff by supporting their educational needs.
	Organisational leadership engagement	Are organisational leaders/managers (e.g. CEOs, executive members) committed/to the implementation effort?		Senior managers: • Encouraged engagement • Ensured follow through with outcomes • Provided resources • Organised implementation
	Available resources	The level of resources available within the organisation to complete the intervention including human (e.g. appropriate staffing levels), financial and technological resources.		Noted that if one team member “was left do his job, the hospital would benefit but it doesn’t have the resources”.
Team-Level Determinants	Structural characteristics	Team size		Workload: participant notes she had “no time” to prepare for the intervention, it “makes up 0.001% of our work”.
		Team turnover/stability		
		Team workload		
	Teamwork	The quality of communication within the team and relationships amongst its members.		“Unless you approach {them} you would get no communication throughout the day”.
	Culture	The norms, values and assumptions of the team, the degree of autonomy given to staff and their perceptions of change.		“Put up and shut up” mind set “…views are valued, sought out in comparison to other multidisciplinary teams I would have been on…like every member is valued and their input is welcomed”.
	Compatibility	Does the intervention fit with existing workflows of the team?		Due to the “pressurised” nature of the ward environment (high patient turnover and poor staffing levels) the compatibility of intervention with the team’s current workload is questionable.
	Available resources	The level of resources available to complete the intervention within the team including human (e.g. appropriate staffing levels), financial and technological resources.		Inadequate staffing impeded staff engagement with the intervention: “we were short staffed, just couldn’t get the time”.
	Local leadership engagement	Are frontline leaders/managers (e.g. consultants, clinical nurse managers) committed and involved in the implementation? Are peer leaders evident?		One senior team member asks to take intervention materials to use with junior doctors at another education session.
	Team efficacy	Does the team believe in their skills and capabilities to implement the intervention successfully?		The team raise concerns regarding lack of training and skills to achieve their developed goals.
Individual-Level Determinants	Self -efficacy	An individual’s belief in their capabilities to implement the intervention and manage its outputs.		One team member indicates that he is capable to contribute more to the team, but his job role does not allow this.
	Individual attitudes	Participants perceptions of the advantage and relevance of the intervention. Is the intervention’s implementation considered a priority or an additional burden in daily practice?		The intervention is “a great way of stopping and reflecting”.

The coding framework enables the collation of various data sources. The data utilised in this wider body of research are presented in Table 3. As data is collected the context coding framework can be continuously updated and adapted by the researcher. This approach aligns with Stake’s [37] guidance on completing a case study which advises that a chronological description of a case is reported. Given the complexity of the construct, using multiple methods facilitates a rich and nuanced understanding of context. However, for some researchers, staffing, funding, and time restrictions may hinder the use of multiple methods and repeated analyses. Therefore, researchers should consider the context and complexity of their project and adapt the framework accordingly.

**Table 3:** Data sources utilised within the context coding framework

Data Sources	Implementation data from wider collective leadership intervention- pre/post survey and interview data
	Observational field notes
	Interview data (implementation focus)
	Annual hospital reports
	Culture audits
	Staff satisfaction survey results

A pre-existing rating criteria developed by Damschroder and Lowery [38] has also been integrated to our context coding framework (Table 4). The ratings reflect the influence (positive, negative, or neutral) and the strength of each contextual feature prior to and during the implementation of the collective leadership intervention. However, additional contextual evaluations may be included as applicable to the research (e.g. multiple periods of data collection as part of a longitudinal evaluation of an intervention).

**Table 4:** Criteria used to assign ratings to constructs-Adapted from Damschroder & Lowery (2013) (http://creativecommons.org/licenses/by/4.0/)

Rating	Criteria
−2	The construct is a negative influence in the organisation, and/or an impeding influence in implementation efforts. Majority of participants describe how the construct manifests in a negative way by describing explicit examples.
-1	There is a mixed effect but overall the construct is noted to be a negative influence in the organisation, and/or an impeding influence in implementation efforts. Participants describe how the construct manifests in a negative way but without concrete examples or sufficient information are given to make an indirect inference of a negative effect.
0	A construct has a neutral influence if it appears to have a neutral effect (participants contradict each other) or there is no evidence positive or negative.
+ 1	There is a mixed effect but overall the construct is noted to be a positive influence in the organisation, and/or a facilitating influence in implementation efforts. Participants describe how the construct manifests in a positive way but without concrete examples or sufficient information is given to make an indirect inference of a positive effect.
+ 2	The construct is a positive influence in the organisation, and/or a facilitating influence in implementation efforts. Majority of participants describe how the construct manifests in a positive way by describing explicit examples.

### Data analysis

**Fig. 2:**
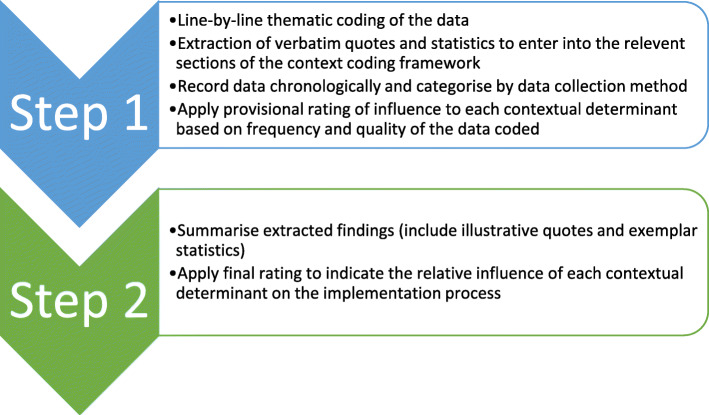
Process of Application

Comparable to the extant literature which derives analytical codes from CFIR constructs [38–40], the context coding framework operates as a codebook during the evaluation process. Consistent with the CFIR Guide (available at www.cfirguide.org), the context coding framework incorporates detailed definitions and examples to ensure the consistent interpretation of codes. However, unlike previous analysis techniques, the framework employs a blended evaluation approach that combines aspects of rapid evaluation and in-depth analysis. Aligned with previous rapid evaluations, the context coding framework provides a visual display that succinctly collates multiple data sources [40, 41]. This method enhances the simplicity of applying Damschroder and Lowry’s rating process [38]. Using an iterative approach, data collection and analysis are concurrent rather than successive [37, 42]. Comparable to more in-depth qualitative evaluations, rather than summarising the data from the outset [40], the framework formally codes each data source as a first analytic step. Although the flexibility of the framework offers remit for an inductive, exploratory analysis, unlike more traditional qualitative approaches, a deductive template is predominantly used to structure the ‘real-time’ analysis of data as it is collected [43]. Consequently, the framework prompts the assessment of potentially influential contextual determinants from the outset. For example, by appraising the construct team workload prior to implementation, researchers can ensure the appropriate resources are available to strengthen the likelihood of implementation success (e.g. need for increased staffing/ researcher support). Thus, the context coding framework offers a structured approach to support intermediary rapid and in-depth evaluations which generates actionable and detailed findings. Figure 2 outlines the process of applying the context coding framework.

#### Step 1

Each data source was analysed by one researcher (LR) using line-by-line thematic coding as outlined by Braun and Clarke [44]. As data emerged, statistics and verbatim quotes were extracted and entered in the relevant section of the context coding framework. Data within each section of the framework was categorised by method of data collection (i.e., observations, organisational reports, interviews, survey data) and recorded chronologically. By employing a triangulation of qualitative research methods a rich, detailed, comprehensive understanding of the context was achieved, enhancing the credibility of the analysis [45, 46]. To manage the large volume of interview data, positive and negative participant quotes were categorised separately to assist with the analysis of each framework domain. Reflecting the frequency and quality of the coded data, LR gave a provisional rating of influence to each contextual determinant within the framework. As new data emerged this rating was discussed and deliberated with researchers familiar with the data and the settings (ADB and EMC were involved with data collection and analysis for the wider body of research evaluating the effectiveness of the collective leadership intervention) and ratings were updated as required. Although double coding of all data sources would be preferable, this approach was unfeasible due to the large volume of data collected and the limited resources available to this study. However, ADB double coded 10% of observation and interview transcripts and all authors engaged in regular coding checks during team meetings throughout the evaluation process (Table 5). Additionally, the dependability of these findings was further heightened by maintaining an audit trail of all decisions and changes in thought processes throughout the analysis process [46].

**Table 5:** Techniques used to enhance the scientific rigour of the context coding framework

Trustworthiness	Application in case study	Additional application strategies
Credibility	Prolonged engagement: continuous data collection to provide sufficient understanding of the context. Triangulation of data: use of multiple methods to develop a detailed understanding of the context. Reflexivity: authors discussed their biases and assumptions throughout the evaluation process. Deviant case analysis: data that did not correspond with emerging coding patterns were included	Member checking: verifying findings with participants
Dependability	Coding checks were completed during team meetings throughout the evaluation process. Audit trail: decisions on rating adjustments were recorded throughout the evaluation process	Double coding: more than one researcher independently assesses the data and the consistency of the rating is compared

#### Step 2

Following the completion of data collection at each phase (prior to implementation and post-implementation), the extracted findings were summarised by LR under each section of the context coding framework. This summary included illustrative quotes and exemplar statistics. Reflecting this summarised narrative, a final rating was applied by the authors to indicate the relative influence of each contextual determinant on the implementation process. We acknowledge that this is a subjective rating based on mostly qualitative data, thus, to achieve actionable results while maintaining scientific rigor, Lincoln and Guba’s [46] trustworthiness criteria were applied to enhance the credibility and dependability of approach. Table 5 presents the techniques applied throughout the evaluation process and identifies additional strategies that researchers may employ to further enhance the scientific rigour associated with the framework’s application.

### Ethical approval

Favourable ethical opinion for the research has been obtained from the University College Dublin Research Ethics Committee (ref: HREC-LS-16-116,397) and participating hospital sites. All participants provided written informed consent and the data collected was inputted into the context coding framework with all potentially identifiable characteristics removed to maintain anonymity.

## Results

When data collection and analysis were complete, data were summarised to give an overarching narrative of the contextual conditions revealed. By employing the context coding framework, the structure enabled innovative insights to be generated throughout the implementation process which facilitated a transparent account of context to be documented and monitored over time.

### Considering the influence of context prior to implementation

Prior to implementation, the context coding framework supported the identification of potentially influential contextual determinants requiring consideration. Table 6 exemplifies one feature of the framework at an organisational and team level to illustrate the value inherent in this approach. Although a facilitative organisational culture was portrayed within hospital documents, survey and interview data obtained at a team level highlighted that a traditional hierarchical context existed that would likely impede implementation success. Some team members felt intimidated by colleagues while others suggested that silo working was apparent with some consultants *“dismissive of any other disciplines”.* This context suggests that the introduction of the collective leadership intervention was timely However, the need to gain and sustain consultant support throughout the implementation process was apparent as consultants appeared to be highly influential in determining team operations and processes; *“what they say goes”.*

**Table 6:** Context coding framework pre-implementation: Culture (Case A)

System Level	Characteristic	Site Description	Construct Rating
**Organisational-Level Determinants**	Culture: *The norms, values and assumptions of the organisation, the degree of autonomy given to staff and their perceptions of change*	• Hospital reports suggests a culture characterised by openness, trust, and inclusion	**+ 2** Construct has a positive effect that may facilitate implementation
**Team-Level Determinants**	Culture: *The norms, values and assumptions of the team, the degree of autonomy given to staff and their perceptions of change.*	• Survey data suggests that there is a hierarchical culture within the team in which staff sometimes feel unheard, unable to speak up, and isolated. 16% also agreed that they feel intimidated by team members' behavior • Interview data also highlights a hierarchical culture: ◦ Control- “the consultants are in charge, what they say goes, the consultants make demands”-some noted to “push the boundaries” ◦ Silo working- “We’re just so individual…it is quite siloed”, “Everyone just kind of looks after themselves” ◦ Staff “afraid to open your mouth” to a senior member of staff, this “fear factor” is recognised as impacting the reporting culture within the team ◦ Suggested that senior doctors are “dismissive of any other disciplines”, “never ask my opinion” • Despite the hierarchy discussed by most participants, some team members are “satisfied” with the team and feel that “everyone is listened to”	**−2** Construct has a negative effect and may impede implementation

The context coding tool can also identify variations in context across settings throughout an intervention’s implementation. As mentioned in the introduction, the collective leadership intervention was implemented in four healthcare teams. While Case A is documented as demonstrating a hierarchical team structure (Table 6), characterised by consultant control, silo working and *“fear”,* this was not representative of the culture within each of the four teams. By applying the context coding framework to another case with similar structural characteristics, a very different culture may be effectively identified. For instance, aligned with the organisation’s public documents and reports, prior to implementation, Case B characterised its culture as *“open”* with a *“multidisciplinary focus”* in which *“every member is valued, and their input is welcomed”.* When introducing an initiative to multiple sites, the rich information gained from applying the context coding framework helps to inform researchers of the differing contextual forces that may exist in each setting throughout an implementation effort. This should ultimately support the likelihood of implementation success across sites as researchers can tailor the implementation strategies they employ.

### Understanding the dynamics of context

Context is made up of multidirectional forces [6, 34]. Its constructs are *“suspended in a complex web of relevance to and relationship with other constructs”* [47]. The developed context coding framework facilitates the exploration of these relationships and the dynamism of the concept as each construct is collectively assessed over time. For example, Tables 7 and 8 confirm the association between the culture of Case A, and the relationships and communication of team members within the organisation. Table 8 clearly identifies the effects of this hierarchical environment (Table 6) which is depicted by inadequate communication and hostility across and within professional groups. The framework also highlights once again the possible disconnect between senior management and frontline staff. Numerous opportunities are listed for team members to voice their frustrations at an organisational level (Table 7), however, these are not operationalised to support staff within their local department (Table 8). Alternatively, it may be interpreted that while the wider organisation is characterised as a supportive environment, the participating team may be an isolated, divergent case. Therefore, the variation in how the organisation at a meso level and the team at a micro level understand and illustrate their context is clearly evident within this framework.

**Table 7:** Context coding framework pre- and post-implementation: Networks and Communication (Case A)

System Level	Characteristic	Site Description	Construct Rating
**Organisational-Level Determinants**	Networks and communications: *The quality of communication within the organisation and relationships amongst its members*.	**Pre implementation**	
		• Hospital documents report that various forms of communication are used within the hospital as a whole (town hall meetings, hospital newsletter, informal meetings with the CEO) and at a local level (staff meetings, WhatsApp groups). • However, it is acknowledged that most staff do not have a digital identity signifying that their ability to receive information is dependent on their relevant line managers sharing information. • Hospital documents report a high level of “camaraderie” among staff which is compared to a “family like feeling” in each department.	**+ 1** Construct has a mixed effect but predominantly positive and may enhance implementation
		**Post implementation**	
		• Hospital documents report that various forms of communication are used within the wider hospital (town hall meetings, staff information sessions, communication steering committee) • From the national survey data *relationships* appear to be strong within the hospital with 80% agreeing that they receive support from colleagues • However, *communication* appears to be an issue within the hospital ◦ Although communication appears to be satisfactory within their team (67%) and with their line managers (65%), frontline staff report a lack of inclusive decision making with only 50% feeling that they have input into decisions that affect their work ◦ Communication with senior management appears unsatisfactory for most staff (42% satisfied) with only 45% content with the feedback mechanisms within the organisation	**0** Construct has a mixed, neutral effect

**Table 8:** Context coding framework pre-and post-implementation: Teamwork (Case A)

System Level	Characteristic	Site Description	Construct Rating
		**Pre implementation**	
**Team-Level Determinants**	Teamwork: *The quality of communication within the team and relationships amongst its members.*	• From the survey data it is evident that the team do not have a forum to meet regularly, share information and provide feedback, with potential tension acknowledged between disciplines with only 40% agreeing that the doctors and nurses collaborate well. • From the interview data *communication* suggested as an issue- “something that needs to be worked on hugely”: ◦ Inter-professional communication poor (“communication gets muddled up a lot”). ◦ Mixed views in relation to the communication between front-line and senior management: noted to be a lack of feedback but acknowledged that communication has improved since the appointment of a new CEO who is commended for “praising where praise is due”. ◦ The personalities of people “in senior positions” suggested as impacting communication which leads to “a bit of clashing” • From the interview data regarding *relationships* tensions were revealed within the team: ◦ Intra-professional tension: conflict in relation to the priorities of junior vs more senior members of staff: “…they are more about paperwork than the patient” ◦ Inter-professional tension: between disciplines; “I’m here to nurse, you’re here to do everything else”, “I have come across a nurse who is afraid of a doctor” ◦ Management and frontline tension: “Sometimes we are underappreciated by management”, “people above me…are meeting every day of the week. I don’t know what people do be meeting about but there are meetings every day, every hour of the week”-which is not fed back to staff. • Relationships suggested to be impacted by the busyness of the ward, rotation of staff, and “personalities” within the team (one team member described as “a bit difficult” by some participants which impacts their ability to speak up) • Few participants describe the relationships among the team positively characterising them as “open” and encouraging “mutual respect”	**-2** Construct may be an impeding influence on implementation
		**Post implementation**	
		• From the observational data *communication* suggested as an issue but improving- ◦ Open communication impacted by the fear culture within the team-impacting team member’s ability to speak up ◦ Written and verbal communication remains poor e.g. some staff report being the “middle-man” passing information between disciplines, noted that poor documentation is “part of the culture” ◦ Feedback from senior management noted to be unsatisfactory with information not being “filtered down” to staff on the ground ◦ However, sense that communication is improving between team members (e.g. doctors using the nurses’ first names) ◦ Improvements in communication recognised as being associated with improved relationships- “getting to know each other better” leading to greater ability to “voice {their} opinion quicker” • From the interview data *communication* within the team is noted to be improving but some issues remain ◦ Communication is noted to be “disjointed” among some disciplines. It is suggested that although communication is good between most team members some consultants wouldn’t “value your opinion” and would change the plan of care without consulting the wider multidisciplinary team. ◦ During the implementation of collective leadership intervention team members report that communication has “opened a bit more” with participants reporting that they feel “allowed to say {their concern/opinion} and voice it”. This is supported by senior medical professionals who note that “anybody now can talk to you… there is no limit. There’s nothing between us”, “you have a bit more ear” in relation to listening to other disciplines within the team. • From the observational data regarding *relationships* some tensions were revealed among the team members, but this is also improving: ◦ Inter-professional tension at times between team members across the multidisciplinary team. ◦ Noted that the team can “have banter now”, with one team member who was previously “standoffish”, “making an effort” with staff • From the interview data *relationships* were acknowledged as improving. ◦ Participants suggested that there was greater “camaraderie” among team members. ◦ Participants noted that the sessions enabled participants to get to know each other on a more personal level which “brought down some barriers” and allowed staff to see each other “in a different light”, making staff more approachable; “*you can say something”* • Relationships were noted to be dependent on the personalities of team members, a participant’s role within the team (health and social care professionals feel more “removed”) and the continuous rotation of staff	**−1** Construct has a mixed effect but predominantly negative and may impede implementation

### Monitoring contextual dynamics over time

The framework also enables contextual factors to be monitored longitudinally, highlighting not only the impact of context on the intervention’s implementation but also the intervention’s possible influence on the surrounding context. Table 8 documents that while communication remains “*disjointed”* between some professions, there is consensus that communication has *“opened a bit more”* due to improved relationships among the team. Following the introduction of the collective leadership intervention, informal relationships have been established, meaning staff perceive each other *“in a different light”*. This has enabled team members to approach senior colleagues more comfortably as they feel *“allowed to say it {their concern/opinion} and voice it”*. It also appears to have broadened the narrow perspectives of senior members who observe that following the implementation of the intervention *“anybody now can talk to you…there is no limit. There’s nothing between us”*. Therefore, the complex interplay between the intervention and the context in which the change is implemented is clearly illustrated over time within the context coding framework.

### Informing the selection of implementation strategies

By enabling the influence of context to be mapped over time, the context coding framework also assists researchers in selecting and tailoring the appropriate implementation strategies for their context of study. Implementation strategies are defined as the *“methods or techniques used to enhance the adoption, implementation, and sustainability of a clinical program or practice”* [48]. As outlined previously, consultants were identified as the dominant leaders of Case A prior to implementation. By appraising this information researchers can ensure their implementation effort includes educational sessions or meetings tailored to these individuals, so they are fully informed of the intervention’s benefits. By gaining their support, this will likely impact the adoption of the collective leadership intervention due to the reported influence they have on the team.

Additionally, the context coding framework can aid in clarifying the association between contextual determinants and change strategies as a transparent account of this interaction can be reported. For example, consultant engagement can be monitored throughout the implementation of the collective leadership intervention. If this remains poor and is observed to be impacting adoption, the researchers could tailor their strategy and perhaps involve the executive board to provide incentives for participation or disincentives for poor engagement. By continuously monitoring the implementation strategies employed, the context coding framework, assists researchers in documenting the effects of these strategies on the local context, aiding in their refinement, modification or adoption of alternative approaches, as required.

## Discussion

This paper has described the value of using our developed context coding framework as a methodology to explore and understand an often-neglected area of research within the field of implementation research, the study of context. Despite the acknowledged influence of context on implementation processes [10, 11, 13, 49, 50], it is reported that historically studies tend to be *“acontextual”* in character or represent context as a stable entity or a unidirectional set of influences [30, 34]. However, this case study illustrates the benefit of using the context coding framework as it offers a means for exploring the active role of context and the dynamism of the concept *“in a world on the move”* [51]. It also responds to previous literature [17–20], by providing a standardised approach to improve the reporting of context during implementation.

By accounting for the complexity of context, Tsoukas [52] suggests that the researcher is providing a meaningful contribution as organisational realities are exposed rather than constrained by a simplified, limited representation. However, this complexity, although powerful [12–14], is purported to impede the study of context [53]. Through developing a standardised approach, the context coding framework assists researchers by making the implicit aspects of context more observable. By considering these multifaceted contextual conditions together across system levels, an interpretable, transparent understanding of context can be elucidated, subsequently advancing the field of implementation research.

Although some studies recognise the multifaceted nature of context [20, 34, 54], this is not a universal perspective. Nielsen and Miraglia [55] describe the distinction between omnibus (stable characteristics of an organisation) and discrete contextual factors (changes taking place throughout implementation). Etymologically, the term context means to knit together or to make connections [14]. Therefore, the context coding framework challenges the simplicity and dichotomy of presenting contextual factors as omnibus or discrete. As illustrated within this case study, contextual factors cannot be depicted as stable, isolated entities. It is the dynamic relationships between these complex constructs rather than their individual forces that have a significant influence on implementation processes. Fitzgerald and McDermott [54] suggest that to capture these interactionist aspects of the concept, context needs to be analysed across multiple system levels. In response to the dearth of guidance provided within the extant literature, the context coding framework provides a structure to enable these dynamic and multidirectional interactions to be documented across the health system.

Fitzgerald [56] suggests that developments are needed to progress research from post hoc analyses of context, toward the ability to predict between alternative scenarios. As illustrated by this case study, the context coding framework provides researchers with a method of weighing the possible influence, affect and effect of differing contextual conditions prior to implementation. Using the integrated rating criteria [38], researchers can identify potential ‘receptive’ and ‘non-receptive’ contexts for change and select the most appropriate implementation strategies relevant to the context(s) of study.

Determining the most appropriate implementation strategy to address an identified contextual determinant has been cited as a fundamental challenge of implementation science [57]. Powell et al. [58] provide a list of 73 discrete implementation strategies that can be utilised by researchers to enhance their implementation effort. These strategies are context dependent signifying that the success of a strategy in one context may fail in another. However literature suggests that few studies tailor their implementation strategies to the context of study [57, 59, 60] or apply them to the appropriate system level (e.g. organisational-focused strategy used to address a contextual determinant at the team level) [59, 61]. Aligned with Grol and Bosch’s [60] suggestion, the context coding framework provides a systematic and rigorous method to help clarify the association between contextual determinants and change strategies as a transparent account of this interaction can be reported across system levels. This builds on the work of Waltz et al. [57], who have developed the CFIR-Expert Recommendations for Implementation Change (ERIC) Implementation Strategy Matching Tool. This tool supports researchers in choosing the best implementation strategy to address CFIR-based barriers [57]. By enabling researchers to document an accurate narrative of the context longitudinally, the context coding framework enables the improved use of the CFIR-ERIC Implementation Strategy Matching Tool. By continuously monitoring these strategies over time further refinements and modifications can be conducted depending on their observed effects on the local context which will likely support and enhance implementation success.

While this methodology is useful in understanding context prior to implementation, its value is not limited to a solitary phase of the implementation process. The Greek philosopher Herachitus argues that *“the only thing that is constant is change”*. Therefore, it is vital to monitor the changing context of healthcare when introducing an initiative. Pettigrew et al. [30] concluded that studies predominantly fail to *“allow the change process to reveal itself in any kind of substantially temporal or contextual manner”*. The context coding framework responds to this criticism by offering a means to monitor the local context longitudinally. This makes it a powerful tool for documenting both the impact of context on the implementation effort and the effect of the intervention on the local context over time. Additionally, although the CFIR conceptualises context as a multilevel construct, our developed framework accounts for the ongoing change that occurs within the health system. Therefore, the context coding framework builds on the CFIR by enabling this ongoing measurement of context and the assessment of its influence longitudinally, across system levels.

### Limitations

There are some important limitations to consider when using the context coding framework. *“To understand anything well we must grasp it in its context”* [12]. However, it is difficult to grasp all features of every context in one framework. While this method offers a means for studying the active role of contextual determinants, researchers are cautioned against drawing a boundary around the phenomenon by only including the contextual factors listed herein. Although the context framework has been informed by the extant literature and the comprehensive nature of the CFIR, it is also shaped by the researchers’ experience of the case studies employed. Therefore, it is recommended that when using the framework, researchers are open and attentive to the inclusion of additional contextual factors that are relevant to their context of study, adapting the framework accordingly. Research is also acknowledged to be *“a product of its time”* [14]. Although the context coding framework provides a transparent account of the dynamism of context longitudinally, it is important that the timing of data collection is considered and documented. With the passage of time the meaning of these contextual constructs and their influence can change.

Additionally, while the framework enables the collation of multiple data sources and offers a high-level overview of implementation influence (positive, negative, neutral), further analysis is required to reveal the mechanisms through which context influences implementation success. However, by using the context coding framework researchers will obtain a comprehensive understanding of the data which will likely accelerate any additional in-depth analyses. We recommend applying Proctor et al.’s [62] implementation outcomes during the next phase of analysis to advance researcher knowledge of the interrelationships between implementation outcomes and contextual determinants.

Furthermore, we acknowledge that this approach is likely to be resource intensive, given the data required to ensure a rich understanding of the complex construct of context. Although the cost of the framework was not formally assessed, it is worth detailing the labour associated with application of this approach in the wider research project. LR invested 100% of her time to this project over a two-year period as part of her doctoral studies. The researcher’s involvement in the framework’s development and their familiarity with the data sources (involved in data collection and transcription), likely expedited the analysis process. However, while future researchers may require training in line-by-line coding or other forms of qualitative analysis, learning to populate the tool requires limited guidance. Larger teams will also have greater capacity to distribute workload to account for any competing priorities. Therefore, before applying this method, researchers should consider the compatibility of the approach with the project aims, team logistics, and project deadlines. Adapted versions of the framework may also be used if researchers are interested only in specifics dimensions of context.

### Recommendations for future use

Aligned with Chambers [63], we recommend the need to use shared measures of context consistently over time in diverse settings to comprehensively understand the complexity of the construct and its influence. This research has addressed an identified gap in implementation science methodology by creating a resource suitable for both researchers and non-researchers (e.g. healthcare professionals, policy makers) to investigate an underdeveloped area of study, our understanding of context in implementation research. It is suggested that by using the context coding framework as a standardised tool across multiple settings, we will likely gain an enhanced knowledge of the construct from multiple perspectives. This will offer valuable guidance to researchers during their future implementation efforts while also providing insight into how context is perceived from differing vantage points (e.g., researcher vs non-researcher perspective, or between healthcare professions). If we do not begin to use a standardised approach consistently, our ability to understand and capture the dynamism of context and its multiple levels will remain severely limited.

## Conclusion

This study described how the context coding framework was developed, outlining the benefits and limitations of employing this approach. The illustrative case study highlighted the value of using this approach as a method to (a) highlight the influence of context prior to implementation, (b) understand the dynamics of context throughout the implementation process, (c) monitor these contextual dynamics longitudinally and (d) inform the selection of the most relevant implementation strategies. This research advances the field by providing a practical methodology that focuses on an overlooked area of implementation science, the study of context. By helping to appropriately assess and report context, the robustness and learning acquired from implementation research will be enhanced, aiding in the translation of evidence-based healthcare interventions into routine practice. We welcome comments and critiques that will refine and further enhance the context coding frameworks utility to progress implementation research and practice.

## Data Availability

Data sharing is not applicable due to privacy/ethical restrictions.

## Abbreviations

CFIR: Consolidated Framework for Implementation Research
ERIC: Expert Recommendations for Implementing Change
